# Potential Explanations for Conflicting Findings on Abrupt Versus Gradual Smoking Cessation: A Population Study in England

**DOI:** 10.1093/ntr/ntab239

**Published:** 2021-11-18

**Authors:** Claire Garnett, Jamie Brown, Lion Shahab, Tobias Raupach, Nicola Lindson

**Affiliations:** 1 Department of Behavioural Science and Health, University College London, London, UK; 2 Spectrum Consortium, London, UK; 3 Department of Cardiology and Pneumology, University Medical Centre Göttingen, Göttingen, Germany; 4 Nuffield Department of Primary Care Health Sciences, University of Oxford, Oxford, UK

## Abstract

**Introduction:**

Observational and trial evidence conflict on the efficacy of two contrasting behavioral approaches to quitting smoking—gradual and abrupt. Observational data suggest an abrupt approach to quitting is superior to a gradual approach, whilst trials show no difference. One potential explanation is self-selection in observational data, whereby people can choose their quit approach, and those who find it harder to quit may be more likely to choose a gradual quit approach. This study aims to investigate potential explanations for these conflicting findings.

**Aims and Methods:**

This study aims to investigate potential explanations for these conflicting findings. We used observational data from a nationally representative sample of adults in England from November 2006 to February 2020 who reported smoking and had made at least one quit attempt in the past year (*n* = 21 542). We used logistic regression models to assess the association between abrupt versus gradual quit attempts and quit success, adjusting for sociodemographic, smoking, and quit attempt characteristics.

**Findings:**

Abrupt, versus gradual, attempts were associated with improved quit success in an unadjusted model (odds ratio = 2.02, 95% CI = 1.86 to 2.19). This association remained after adjusting for a broad range of relevant confounders (odds ratio = 1.75, 95% CI = 1.59 to 1.93).

**Conclusions:**

Among a representative sample of adults who had smoked and made a quit attempt in the past year, there was evidence of an association between abrupt attempts and quit success before and after adjusting for relevant confounders. This suggests that the differences in quit success seen between abrupt and gradual quit attempt types are not completely driven by self-selection in observational data.

**Implications:**

We investigated explanations for conflicting findings on the efficacy of gradual versus abrupt approaches to quitting smoking between trial and observational data. Despite adjusting observational data for sociodemographic, smoking, and quit attempt characteristics, an association between abrupt quitting and quit success remained. Therefore, differences in quit success were not completely driven by the self-selection of a gradual approach by people who found it especially difficult to quit or differences in the use of quitting aids. However, characteristics adjusted for were limited by the data available, and future research should continue to investigate the difference in findings across study types to inform cessation support.

## Introduction

Attempts to quit smoking are typically classified as either gradual (cutting down before the attempt) or abrupt (making the attempt without cutting down first).^1^ Historically, the standard way people who smoke have been advised to quit is abruptly. However, survey data suggest quitting gradually, by reducing cigarette consumption before stopping smoking completely, is an approach that people who smoke find appealing.^1,2^ As a result, the relative efficacy of the two approaches to smoking cessation has been investigated using both randomized controlled trials (RCTs) and observational studies; however, conclusions drawn from RCT and observational data have conflicted.^1,3,4^ A Cochrane systematic review and meta-analysis of 22 RCTs found moderate certainty evidence that both abrupt nor gradual quitting resulted in similar quit rates at long-term follow-up^4^; whereas survey data has generally found a benefit of quitting abruptly over gradually.^1,5^

A recent editorial summarized the existing evidence and hypothesized explanations for the lack of consensus across the literature.^6^ Four explanations emerged regarding real-world quit attempts (as opposed to quit attempts made as part of an RCT). The first was “*consistency of approach across multiple quit attempts*”: people who smoke may use more than one approach to quitting and may be more likely to frame and label their quit attempt as abrupt if it was successful in observational data, even if they did reduce their smoking at some point in the run up to the attempt. Whilst in trial data, the quit attempt approach is assigned and labeled by the researchers, not allowing for reframing of an attempt approach based on its success. This explanation can be explored using observational data by assessing whether those adults who smoke and make multiple attempts switch between the two quit attempt approaches, and if there are higher quit success rates among those who always make abrupt attempts (ie, definitely not a case of the approach being labeled as abrupt based on whether it was successful given that at least one past abrupt attempt has failed by definition) than those who either always make gradual or switch between the two approaches.

The second explanation was “*self-selection*” whereby those people with certain sociodemographic and smoking characteristics, who generally find it harder to quit (eg, those with mental health problems, lower socioeconomic status, higher levels of addiction^7^), may favor and choose to quit gradually in observational studies. Whilst in RCTs the individuals do not have a choice over their quit attempt approach and may not get the approach that they prefer. This explanation can be explored using observational data by adjusting for the relevant self-selection variables to assess the association more directly between abrupt quit attempts and quit success.

The third explanation was “*use of evidence-based quitting aids*” where in observational data people who smoke and make gradual quit attempts may use fewer, or less effective, evidence-based aids (eg, pharmacotherapies or behavioral support) than those who report abrupt quitting. In RCTs, participants may be given instructions about the use of quitting aids, or if it was not assigned, the use of aids would likely be equivalent across groups due to randomization. This explanation can be explored using observational data by adjusting for use of evidence-based aids when assessing the association between abrupt attempts and quit success.

The fourth explanation was “*discrepancy between trial and real-world quitting methods*.” People who smoke and make gradual quit attempts may use less effective behavioral reduction methods than those implemented in RCTs. Although evidence of the relative efficacy of different smoking reduction approaches is limited, it could be that some methods work better than others based on things like the thresholds set for reductions in pre-quit smoking behaviors; the duration of the pre-quit reduction period; and whether a quit date was set.^4^ This explanation can only be investigated if the observational data collects detailed information on the exact methods of the quit attempt approach used.

Testing these hypothesized explanations could provide greater understanding of the basis behind the difference in evidence between trial and observational data, and insight into how to maximize the potential of smoking cessation methods. This in turn could enrich the evidence-based options available to smokers wanting to quit. Therefore, the aim of this paper was to use observational data from the Smoking Toolkit Study (STS)^1^ to investigate the first three explanations proposed above (there was a lack of sufficiently detailed information on quit attempt approach methods to investigate the fourth explanation). A paper using data from the STS, published in 2019, found evidence that attempting to quit abruptly was independently associated with an increased quit success rate.^1^ The 2019 study used data from between 2006 to 2016, and respondents were people who smoked at baseline and had attempted to quit between baseline and 6-month follow-up (ie, quit success assessed prospectively), therefore, limiting the available sample size. This current study will use data from respondents who had smoked in the past-year at the time of the baseline survey and had made a quit attempt in the previous 12 months, allowing for a substantially larger sample size. Using this dataset, we answered the following research questions:

## Research Questions

*Confirmation of the findings of STS prospective data analysis*

1. Are abrupt (compared with gradual) quit attempts (at the most recent attempt) associated with improved quit success rates among adults who smoke in England who made a quit attempt in the past 12 months, when retrospectively identifying quit attempts from a larger sample?

*Testing the “consistency in approach across multiple quit attempts” explanation*

2. Do adults who smoke in England, and have made at least two quit attempts in the past 12 months, switch between abrupt and gradual quit attempts (consistent, ie, “all abrupt” or “all gradual” or inconsistent, ie, “abrupt and gradual”)? If so, do people whose quit attempts are “always abrupt” experience improved quit success rates compared with people who make “any gradual” (ie, “all gradual” or “abrupt and gradual”) quit attempts?

*Testing the “self-selection” and “use of evidence-based aids” explanations*

3. Do sociodemographic, smoking, or quit attempt characteristics differ between people who report abrupt versus gradual quit attempts (at their most recent attempt)?

*Associations with quit attempt approach or success*

4. Are any sociodemographic, smoking, or quit attempt characteristics independently associated with abrupt versus gradual quit attempts (at their most recent attempt)?
5. Are abrupt (compared with gradual) quit attempts independently associated with improved quit success rates (at their most recent attempt) after adjusting for relevant sociodemographic, smoking, and quit attempt characteristics?

## Methods

### Design and Study Population

The STS is an ongoing, monthly, population survey in England. The STS consists of cross-sectional household surveys of nationally representative samples of 1700–1800 adults (aged 16+) in England.^8^ The study sampling is a hybrid of random probability and simple quota. Full details of the study sampling methods are described in full elsewhere.^8^

This study used data from November 2006 until February 2020 from adults aged 16+ who were past-year smokers (including people who were currently smoking and who had quit recently) and had made at least one quit attempt in the past 12 months (ie, quit success assessed retrospectively). For research question 2, only respondents who reported making at least two quit attempts in the past year were included in the analyses. This subsample of respondents was then coded as either consistent (always made the same type of quit attempt; abrupt or gradual) or inconsistent (had used both an abrupt and a gradual quit attempt in the past year), see Table 1.

**Table 1.** Definitions of (1) “Always Abrupt” Versus “Any Gradual” and (2) “Consistent” Versus “Inconsistent” and Examples

Sample	The whole sample (regardless of the number of quit attempts made in the past year)		Only those who made at least two quit attempts in the past year	
	“Always abrupt”	“Any gradual”	Consistent	Inconsistent
	All quit attempt/s made using an abrupt approach	Any/all of the quit attempt/s made using a gradual approach	Always used the same approach (always gradual or always abrupt)	Used both an abrupt and gradual approach
	*If multiple attempts made*			
	Would be consistent	Could be either consistent or inconsistent		

Examples: (1) A respondent who only made one attempt and that attempt was abrupt would be “always abrupt” and have no classification for consistent versus inconsistent. (2) A respondent who made two gradual attempts would be “any gradual” and consistent. (3) A respondent who made one abrupt and one gradual attempt would be “any gradual” and inconsistent.

### Measures

All measures were asked of the respondents during a single survey but not all measures were included in every wave. Full details of all measures, including during which waves the measures were assessed, are listed in Supplementary File 1. Complete cases for the relevant variables were used in the analyses for research questions 1, 2, and 3. For research questions 4 and 5, only those measures consistently asked from the start of the STS (age, sex, social grade, time to first cigarette, strength of urges, use of aids, time since the start of quit attempt, number of quit attempts, quit success, and quit approach type) were included to maximize sample size for the multivariate models, and motivation to quit was dropped as it was only asked of current smokers.

Sociodemographic characteristics assessed were: age (16–24/25–34/35–44/45–54/55–64/65+); sex (female/male); social grade (AB/C1/C2/D/E); annual household income (<£11 499/£11 500–17 499/£17 500–39 999/>£40 000); ethnicity (minority ethnic group/white); highest educational qualification (post-16/pre-16); employment status (full-time job/not); marital status (married/not); children in household (present/absent); housing tenure (owner occupied/other); sexual orientation (heterosexual/not heterosexual); disability (no/yes), and mental health diagnoses (no/yes).

Smoking characteristics assessed were: time to first cigarette (as an indicator of cigarette dependence: more than 60 min/30–60 min/6–30 min/within 5 min^9^); strength of urges (none/slight/moderate/strong/very strong/extremely strong); motivation to quit smoking (using the Motivation to Stop Scale,^10^ continuous score from 1 to 7; only asked of respondents who reported currently smoking at the time); and number of quit attempts in past 12 months (1/2/3 or more).

Quit attempt characteristics (relating to serious quit attempts in the past 12 months) assessed were: quit success (unsuccessful/successful; where successful was defined as a successful quit attempt, ie, any respondent who reported having attempted to quit in the past 12 months and was “still not smoking” at the time of the survey); quit approach at their most recent attempt (gradual [“cut down first”]/abrupt [“stopped without cutting down”]); use of evidence-based aids during their most recent quit attempt (no/yes); and time since the start of their most recent quit attempt (last week/between a week and a month/1–2 months/2–3 months/3–6 months/6–12 months). If evidence-based aids were used in their most recent quit attempt, then respondents were asked what type of aids were used: varenicline; bupropion; nicotine replacement therapy (NRT); e-cigarettes, and face-to-face behavioral support (where the reference category was no use for each aid type). If NRT was used in their most recent quit attempt, respondents were asked if they had used over-the-counter or prescription NRT.

If respondents had made multiple quit attempts in the past 12 months, their quit approach (gradual/abrupt) was also assessed for their second and third most recent quit attempt. All respondents were defined as either “always abrupt attempts” or “any gradual attempt” regardless of the number of quit attempts made in the past year, see Table 1.

### Analyses

All analyses were conducted in R. The analysis plan was pre-registered on the Open Science Framework (https://osf.io/64qsc/), although a number of subsequent changes were made to the pre-registered plan, which are detailed in Supplementary File 1.

#### Confirmation of the Findings of STS Prospective Data Analysis

A binary logistic generalized linear model (GLM) assessed the univariable association between quit success and abrupt versus gradual most recent quit attempt among adults in England who had smoked in the past year and had made a quit attempt in the past 12 months.

#### Testing the “Consistency in Approach Across Multiple Quit Attempts” Explanation

Of the subsample of respondents who reported making at least two quit attempts in the past 12 months, a new dummy coded variable was created for whether respondents always quit in the same way (consistent) or varied their approach to quitting across attempts (inconsistent). Respondents were coded as “consistent” if all their quit attempts in the past 12 months were of the same type and “inconsistent” otherwise. A 2 × 2 chi-squared test assessed the association between gradual versus abrupt most recent quit attempt and consistent versus inconsistent approach to all quit attempts in the past 12 months.

A binary logistic GLM assessed the univariable association between quit success and “always abrupt” versus “any gradual” quit attempts in the past 12 months among those making at least two quit attempts.

#### Testing the “Self-selection” and “Use of Evidence-Based Aids” Explanations

A series of unadjusted binary logistic GLMs assessed the univariable association between each characteristic and abrupt, compared with gradual, quit attempts at the most recent attempt.

#### Associations With Quit Attempt Approach or Success

A binary logistic GLM assessed the independent association between abrupt, compared with gradual, most recent quit attempts and the relevant sociodemographic (age, sex, social grade), smoking (time to first cigarette, strength of urges, number of quit attempts in past year), and quit attempt characteristics (use of aids and time since start of quit attempt).

A binary logistic GLM assessed the association between quit success rates and abrupt compared with gradual quit attempts, adjusting for the relevant sociodemographic (age, sex, social grade), smoking (time to first cigarette, strength of urges, number of quit attempts in past year), and quit attempt (use of aids and time since start of quit attempt) characteristics.

#### Sensitivity Analyses

Sensitivity analyses were conducted using the “always abrupt” versus “any gradual” quit attempt groups for any models assessing associations with quit attempt approach or quit success.

#### Bayes Factors

Bayes factors (BFs) were calculated using an online calculator (www.bayesfactor.info) to examine whether any nonsignificant results indicated evidence of no effect or data being insensitive to detect an effect, and for significant results, the strength of evidence.^11^ Alternative hypotheses were represented by half-normal distributions. The absolute expected effect size for associations with the abrupt versus gradual quit attempt outcome (research questions 3 and 4) was set to an odds ratio (OR) = 2.0 in the observed direction (ie, OR = 2.0 for observed ORs > 1 and OR = 0.5 for observed ORs < 1; BF_(0,2)_), as differences would likely need to be substantial to be implicated in the association between abrupt quit attempts and quit success. The absolute expected effect size for the quit success outcome (research questions 1, 2, and 5) was set to an OR = 3.0 in the observed direction, based on previous research (BF_(0,3)_).^1^ BFs of at least 3 provide evidence for a difference, BFs less than or equal to 0.33 provide evidence for the null hypothesis of no difference, and 0.33 < BFs < 3 indicate the data are insensitive to detect an effect.^12,13^

#### Ethics

Ethical approval for the STS was granted by the UCL Ethics Committee (ID 2808/005). The data are not collected by UCL and are anonymized when received by UCL.

## Results

### Participant Characteristics

A total of 21 542 adults who reported smoking in the past year and had made at least one attempt to quit smoking in the past year were included in the analyses. Of these, 54.9% (*n* = 11 826) had made an abrupt quit attempt at their most recent quit attempt and 65.2% (*n* = 14 054) had made a single-quit attempt in the past 12 months. Participants’ sociodemographic, smoking, and quit attempt characteristics are reported in Supplementary Table 1.

### Confirmation of the Findings of STS Prospective Data Analysis

Abrupt quit attempts, compared with gradual quit attempts, were associated with improved quit success rates (18.8% vs. 10.3%; OR = 2.02 [95% CI = 1.86 to 2.19], *p* < .001, BF_(0,3_) > 10 000).

### Testing the “Consistency in Approach Across Multiple Quit Attempts” Explanation

Of the 7488 respondents who made multiple quit attempts in the past 12 months, 70.1% (*n* = 5247) were consistent in their approach across attempts with 52.7% always making an abrupt attempt (*n* = 2765) and 47.3% always making a gradual attempt (*n* = 2482). Of the 29.9% who were inconsistent across multiple attempts (*n* = 2241), 37.9% (*n* = 849) made an abrupt and 62.1% (*n* = 1392) made a gradual attempt at their most recent attempt.

Among these respondents who made multiple quit attempts in the past 12 months, those whose attempts were “always abrupt,” reported improved quit success rates, compared with those who were coded “any gradual” (10.6% vs. 7.9%; OR = 1.39 [95% CI = 1.19 to 1.63], *p* < .001, BF_(0,3)_ = 518).

### Testing the “Self-selection” and “Use of Evidence-Based Aids” Explanations at the Most Recent Quit Attempt

There were significant differences in sociodemographic, smoking, and quit attempt characteristics between people who reported abrupt versus gradual quitting methods at their most recent quit attempts, see Supplementary Table 1. An abrupt quit attempt was more likely than a gradual attempt in younger people, in a higher social grade with a higher annual household income, a higher level of education, and in a full-time job. It was also more common in people of white ethnicity (compared with a minority ethnic group), and in those who were married and whose housing tenure was owner occupied (though the BF indicated that these data were insensitive to detect an effect size set to OR = 2 in the observed direction).

There were no clear relationship patterns between type of quit attempt and either strength of urges to smoke or time to first cigarette after waking. Those who had made 2 or more quit attempts (compared with 1) in the past 12 months were less likely to report an abrupt quit attempt. Of the current smokers in the sample (ie, those who were unsuccessful in any quit attempts in the past 12 months), respondents were asked about their current motivation to quit smoking and those who reported making a gradual quit attempt at their most recent quit attempt had marginally higher levels of motivation to quit smoking.

In terms of quit attempt characteristics, those who started their most recent quit attempt 3–6 months or 6–12 months ago (compared with last week) were more likely to report an abrupt quit attempt. Those who started their most recent quit attempt between a week and a month, 1–2 months, or 2–3 months ago (compared with last week) were less likely to report an abrupt quit attempt. Those who used any type of NRT were more likely to report an abrupt quit attempt and those who used varenicline and bupropion (compared with those who did not) were less likely to report an abrupt quit attempt. The BFs indicated that the data provide evidence for there being no difference in the number of people who used behavioral support or e-cigarettes to aid abrupt and gradual quit attempts.

A sensitivity analysis comparing characteristics between smokers who reported “always abrupt” and “any gradual” quit attempts showed the same pattern of results, see Supplementary Table 2, suggesting that differences seen between abrupt and gradual quit attempts were not because people who successfully quit were more likely to label their quit attempt as abrupt.

### Associations with Quit Attempt Approach or Success

A total of 20 446 respondents had complete cases for the following variables: age, sex, social grade, time to first cigarette, strength of urges, use of aids, time since start of quit attempt, number of quit attempts, quit success, and quit approach type; and were used as the subsample to investigate associations with quit attempt approach or success.

A number of sociodemographic, smoking, and quit attempt characteristics were independently associated with an abrupt, compared with gradual, quit attempt at their most recent quit attempt, see Supplementary Table 3. Making an abrupt quit attempt was independently associated with being older (35–44 compared with 16–24), smoking within 5 min of waking compared with more than 60 min after waking, starting their most recent quit attempt longer ago (between a week and a month, 1–2 months, 2–3 months, 3–6 months, and 6–12 months earlier compared with last week, though the BF indicated that the data were insensitive to detect an effect size set to OR = 2 in the observed direction for between a week and a month). Making a gradual quit attempt was independently associated with being older (55–64 and 65+ compared with 16–24, though the BF indicated that the data were insensitive to detect an effect size set to OR = 2 in the observed direction), lower social grades (C1, C2, D, and E compared with A), smoking within 30–60 min of waking compared with more than 60 min after waking (though the BF indicated that the data were insensitive to detect an effect size set to OR = 2 in the observed direction), having stronger urges to smoke (slight, moderate, strong, very strong, and extremely strong compared with none, though the BF indicated that the data were insensitive to detect an effect size set to OR = 2 in the observed direction for extremely strong), using an evidence-based aid to quit (compared with not), and having made more quit attempts (two and three or more compared with one) in the past 12 months.

After adjusting for relevant sociodemographic, smoking, and quit attempt characteristics, quit success was independently positively associated with making an abrupt, compared with gradual, quit attempt, see Supplementary Table 3.

Sensitivity analyses using the “always abrupt” versus “any gradual” groups showed the same pattern of results for the associations with quit attempt approach and quit success, see Supplementary Table 4.

## Discussion

### Summary of Findings

Among a representative sample of over 20 000 adults in England, who had both smoked and made a quit attempt in the past year, slightly over half (55%) used an abrupt approach at their most recent attempt. Abrupt quit attempts were associated with improved quit success rates compared with gradual attempts, which replicated the previous finding using prospective STS data.^1^ Just over a third of respondents (35%) had made multiple quit attempts in the past year and of those, the majority (70%) were consistent in their approach to quitting across multiple attempts. Those who had always made abrupt quit attempts across multiple attempts in the past year had higher rates of quit success than people who had made any gradual quit attempts.

This study detected several significant independent differences in sociodemographic, smoking, and quit attempt characteristics between those reporting abrupt and gradual quit attempts at their most recent attempt. However, making a successful quit attempt was still more likely among those who had made an abrupt quit attempt (the association persisted, but was attenuated), after adjusting for these observed differences. This suggests that while there are observed differences in terms of self-selection and the use of evidence-based aids, the differences in quit success seen between abrupt and gradual quit attempt types are not solely driven by these. Therefore, based on the characteristics we were able to investigate those who were less likely to be successful at quitting smoking did not appear more likely to choose a gradual approach. However, it may be that differences in unmeasured or residual confounders exist and could explain the differences in quit success rate between abrupt and gradual quit attempts, particularly given that the effect is attenuated in adjusted analyses. The pattern of findings remained the same when all respondents were defined as making “always abrupt” or “any gradual” quit attempts, suggesting that differences seen between abrupt and gradual quit attempts were not because people who successfully quit were more likely to label their quit attempt as abrupt.

The conflicting evidence for the efficacy of gradual and abrupt quit attempts depending on whether observational or trial data are used may be due to several differences between observational studies and trials. First is the matter of choice that exists in observational studies where individuals can choose the quit attempt approach that they prefer, while in trials individuals are assigned to a quit attempt approach, which may or may not be the approach that they prefer. An RCT that investigated participants’ intervention preference found that participants who preferred a gradual approach were significantly less likely to be abstinent one month later compared with those who preferred an abrupt approach,^3^ regardless of whether they were randomized to abrupt or gradual quitting. This suggests that preference does play a role in quit success. However, the findings from this study suggest that the self-selection explanation does not fully account for the differences seen between observational studies and trials, based on the range of important characteristics that we adjusted for.

Second, observational studies (including this study) usually assess quit attempts retrospectively, that is, over the past year, whilst trials tend to assess quit attempts prospectively. Therefore, observational studies usually rely on recall data, introducing scope for bias. Further research is needed to replicate these findings with prospective quit success data.

Third, it may relate to differences in how gradual and abrupt quitting is operationalized in trials and how gradual and abrupt quitting is understood in observational survey data. In some instances, gradual quitting in a trial is clearly specified; for example, gradually reducing tobacco use over a 2-week period before a quit date, reducing smoking by half in the first week and then to a quarter in the second week of an attempt.^3^ Whilst in observational studies, the question and response options could have a much broader interpretation. In this study, respondents were asked if they cut down the amount they smoked before trying to stop completely, which covers any period of reduction and any proportion of reduction. Therefore, it is important to investigate how people interpret these questions in observational studies and to better understand the details of how gradual quit attempts are operationalized in the “real world”. For example, whether people always acknowledge that any reduction in smoking they make before the quit day is part of the attempt in case there are instances where people have reduced their smoking gradually but consider it an abrupt attempt. Further research is also needed to replicate these findings using other methods, such as ridge regression, to estimate the extent to which correlated variables are independently associated with an outcome.

### Strengths and Limitations

This study is the first to investigate potential explanations for the conflicting findings on the effectiveness of abrupt versus gradual smoking quit attempts depending on whether observational^1^ or trial^4^ data is used. A major strength of this study is that the sample were from a large, representative population survey in England and the findings are therefore likely to be generalizable to the population of smokers in England. Whilst RCTs are the gold standard for effectiveness research, there are often unavoidable sources of bias.^14^ This highlights the importance of triangulation, using different methodological approaches with different key sources of potential bias (that are unrelated to each other) to answer the same research question.^15^ Using an observational study design is more reflective of real-world self-selection and quitting methods and provides insight into how people who smoke choose their quitting method, unlike trials which may not generalize to real-world settings as they provide information under ideal circumstances.

A limitation of this study was the difference in the way the smoking characteristics were asked of current (ie, unsuccessful attempts) versus recent ex-smokers (ie, successful attempts). Current smokers were asked about their current smoking behavior whilst recent ex-smokers were asked about their smoking behavior retrospectively (eg, how soon after you wake up did you light up). This introduces biases in the data collection of smoking characteristics that were unequal with regards to quit success. All respondents were asked about their quit attempt characteristics relating to quit attempts in the past 12 months (ie, assessed retrospectively) meaning that there was a reliance on recall data for quit attempt characteristics over the past year, where recall may have differed based on type of quit attempt, introducing scope for bias. Another limitation of this study is that we were unable to investigate the fourth hypothesis, related to the discrepancy between the quitting methods used in trials and the “real-world” due to the data available, as respondents were not asked to provide detailed explanations of their behavioral quitting methods that could be compared with methods used in RCTs.

### Conclusion

In conclusion, among a representative sample of adult past-year smokers who had made a quit attempt in the past year, there were differences in terms of sociodemographic, smoking, and quit attempt characteristics between those making an abrupt, compared with a gradual, quit attempt at their most recent attempt. However, making a successful quit attempt was more likely among those who had made an abrupt quit attempt both before (OR = 2.02) and after adjusting for relevant sociodemographic, smoking, and quit attempt characteristics (OR = 1.75), though there was attenuation after adjustment. This suggests that the differences in quit success seen between abrupt and gradual quit attempt types are not fully driven by self-selection—that people who choose gradual quit attempts are those who generally find it harder to quit—on a range of important sociodemographic and smoking variables, or the use of evidence-based aids. Further research is needed using prospective data and other methods, such as ridge regression, which can assess whether these findings are replicated and estimate the extent to which correlated variables are independently associated with an outcome.

## Supplementary Material

A Contributorship Form detailing each author’s specific involvement with this content, as well as any supplementary data, are available online at https://academic.oup.com/ntr.

ntab239_suppl_Supplementary_Materials

ntab239_suppl_Supplementary_Tables

ntab239_suppl_Supplementary_Taxonomy_Form

## Data Availability

The data underlying this article are available on Open Science Framework, at https://osf.io/b3c4f/.
