# Supplementary material for: Potential Explanations for Conflicting Findings on Abrupt Versus Gradual Smoking Cessation: A Population Study in England
Source: Nicotine Tob Res. 2021 Nov 18;24(4):574–80. doi: 10.1093/ntr/ntab239 (PMC8887587; doi:10.1093/ntr/ntab239)
Supplement: ntab239_suppl_Supplementary_Materials [file ntab239_suppl_supplementary_materials.docx]

# Supplementary File 1

## Measures

### All of the measures were asked of the respondents during a single survey and were available for all survey waves unless otherwise stated.

### Sociodemographic characteristics

- Age (16-24/25-34/35-44/45-54/55-64/65+)
- Sex (male/female)
- Social grade (AB/C1/C2/D/E); an occupational index of socioeconomic position, where grades A, B and C1 indicate managerial, professional and intermediate occupations and C2, D and E indicate skilled, semi-skilled, unskilled manual, and lowest-grade worked or unemployed.
- Annual household income (<£11,499/£11,500-17,499/£17,500-39,999/>£40,000); only available from March 2013 onwards.
- Ethnicity (minority ethnic group/white); only available from March 2013 onwards.
- Highest educational qualification (post-16/pre-16); only available from March 2013 onwards.
- Employment status (no full time job [part-time (29 hours or less) paid job, self-employed, full time student or still at school, unemployed, retired, not in paid work because of long-term illness or disability or for another reason, or housewife/husband]/full time job); only available from March 2013 onwards.
- Marital status (not married: single, separated, divorced or widowed/married); only available from March 2013 onwards.
- Children in household (not present/present); only available from March 2013 onwards.
- Housing tenure (other housing tenure/owner occupied: owned outright or being bought with a mortgage); only available from March 2013 onwards.
- Sexual orientation (not heterosexual: bisexual, gay, lesbian, or prefer not to say/heterosexual); only available from July 2013 onwards.
- Disability (no/yes); only available from July 2013 onwards.
- Mental health diagnoses (no [ref] vs. yes); only available from January 2016 to December 2017.

### Smoking characteristics

### Respondents who were currently smoking were asked about their smoking characteristics in the present tense (e.g. how soon after you wake up do you light up?), whilst those who had quit were asked about it in the past tense (e.g. how soon after you wake up did you light up).

### Time to first cigarette (indicator of cigarette dependence: more than 60 minutes/30-60 minutes/6-30 minutes/within 5 minutes ^11^)

- Strength of urges (none/slight/moderate/strong/very strong/extremely strong); not available for 6 months (May, July, September, and November 2012, and January and March 2013).
- Motivation to quit smoking (continuous score from 1 to 7; using the Motivation to Stop Scale ^12^); only available from November 2008 onwards and only asked of respondents who were currently smoking, therefore not included in analyses involving quit success.
- Number of quit attempts in past 12 months (1/2/3 or more)

### Quit attempt characteristics (relating to serious quit attempts in the past 12 months):

- Quit success (unsuccessful/successful), where successful was defined as any respondent who reported having quit smoking (‘I have stopped smoking completely in the last year’)
- Quit approach at their most recent attempt (gradual/abrupt); where this was assessed with the question ‘Did you cut down the amount you smoked before trying to stop completely at your most recent serious quit attempt?’ and two options: ‘Stopped without cutting down’ (abrupt) or ‘Cut down first’ (gradual).
- If respondents had made multiple quit attempts in the past 12 months, their quit approach (gradual/abrupt) was also assessed for their second and third most recent quit attempt. Respondents were then defined as ‘always abrupt’ if they had answered abrupt for all of the quit attempts they had made in the past 12 months (even if they made only one quit attempt) and defined as ‘any gradual’ if they answered ‘gradual’ for at least one quit attempt in the past 12 months.
- Use of evidence-based aids during most recent quit attempt (no/yes)
- If evidence-based aids were used in the most recent quit attempt, what types of aids were used (where the reference category was no use):
- Varenicline
- Bupropion
- Nicotine replacement therapy (NRT)
- E-cigarettes
- Face-to-face behavioural support
- If NRT was used, type of NRT used (prescription/over-the-counter)
- Time since the start of their most recent quit attempt (last week/between a week and a month/1-2 months/2-3 months/3-6 months/6-12 months)

## Changes to the pre-registered analysis plan

The analysis plan was pre-registered on the Open Science Framework (<https://osf.io/64qsc/>), although a number of subsequent changes were made to the pre-registered plan: i) complete cases were specified for the abrupt quit attempt variable as this variable was necessary for all analyses; ii) the data on NRT were not sufficiently detailed to analyse differences between fast- and slow-acting NRT or combination versus single form; iii) focused on single use of over-the-counter versus prescription NRT (excluding dual use); iv) the ‘self-selection’ and ‘use of evidence-based aids’ explanations were tested using a series of unadjusted binary logistic GLMs instead of omnibus ANOVAs and chi-squared tests, v) daily cigarette consumption was excluded as a measure from the analysis due to limitations in how and when it was measured, and vi) change in how variables were selected for the analyses for the associations with quit attempt approach or success.

## Limitations with the measure of daily cigarette consumption

The pre-registered analyses which included the measure of daily cigarette consumption are reported in Supplementary Tables 5 and 6. Daily cigarette consumption was significantly higher among those who had made an abrupt quit attempt compared with those who had made a gradual quit attempt (12.9 vs. 11.6; OR=1.02, p<.001; Supplementary Table 5). The pattern of results for the independent associations were similar for quit approach type and quit success, with cigarettes per day being independently positively associated with abrupt quit attempts and independently positively associated with quit success (Supplementary Table 6). However, there are issues with using daily cigarette consumption as a marker of addiction as it is confounded with quit attempt approach. Using daily cigarette consumption is likely to be under-estimate addiction level (in a retrospective study) among people who make a gradual quit attempt (compared with an abrupt quit attempt) regardless of their success in quitting, as they may have lower consumption after their quit attempt as part of the gradual quit approach is based on reducing the number of cigarettes smoked per day ^16^. Therefore, we focused on other smoking characteristics to measure addiction in the main analyses: strength of urges and time to first cigarette. These are key indicators of nicotine dependence ^17^ and there is a lack of good evidence to suggest they are confounded with gradual quit attempt approaches in a similar way to cigarette consumption ^6^. We conducted an exploratory sensitivity analysis among respondents who made a quit attempt between 6 and 12 months ago (n=7,924; 37% of the sample) to check whether the pattern of results for these indicators of nicotine dependence remained the same. This sensitivity analysis showed the same pattern of results for strength of urges and time to first cigarette (see Table below) as with quit attempts as recently as last week, suggesting we can have confidence in these as valid measures of nicotine dependence in this study design.

Exploratory sensitivity analysis among respondents who made a quit attempt 6-12 months ago (n=7,924)

|  | | Whole sample | Abrupt quit attempt | Gradual quit attempt | Association with quit attempt type (abrupt vs. gradual [ref]) | |
| --- | --- | --- | --- | --- | --- | --- |
|  | |  |  |  | OR (95% CI) | p-value |
| Time to first cigarette, % (n) | |  |  |  |  |  |
|  | More than 60 minutes [ref] | 32.8 (2,583) | 34.6 (1,625) | 30.2 (958) |  |  |
|  | 30-60 minutes | 15.8 (1,243) | 14.5 (679) | 17.8 (564) | 0.78 (0.69, 0.89) | <.001 |
|  | 6-30 minutes | 32.4 (2,547) | 31.7 (1,487) | 33.4 (1,060) | 0.93 (0.84, 1.02) | .118 |
|  | Within 5 minutes | 19.0 (1,492) | 19.2 (899) | 18.7 (593) | 1.03 (0.92, 1.16) | .586 |
| Strength of urges, % (n) | |  |  |  |  |  |
|  | None [ref] | 18.4 (1,399) | 21.7 (983) | 13.5 (416) |  |  |
|  | Slight | 13.9 (1,058) | 13.6 (616) | 14.4 (442) | 0.93 (0.82, 1.07) | .315 |
|  | Moderate | 41.2 (3,134) | 39.5 (1,790) | 43.8 (1,344) | 0.84 (0.76, 0.92) | <.001 |
|  | Strong | 18.8 (1,427) | 17.7 (802) | 20.4 (625) | 0.84 (0.75, 0.94) | .003 |
|  | Very strong | 5.5 (422) | 5.3 (240) | 5.9 (182) | 0.89 (0.73, 1.08) | .235 |
|  | Extremely strong | 2.2 (167) | 2.3 (105) | 2.0 (62) | 1.15 (0.84, 1.59) | .388 |

## Analyses for associations with quit attempt approach or success

The original analysis plan for the associations with quit attempt approach or success was as follows:

*A binary logistic GLM assessed the independent association between abrupt, compared with gradual, quit attempts adjusting for the sociodemographic, smoking or quit attempt characteristics that were found to differ between people who attempted to quit gradually and abruptly.*

*A binary logistic GLM assessed the association between quit success rates and abrupt compared with gradual quit attempts, adjusting for the sociodemographic, smoking and quit attempt characteristics that were independently associated with abrupt quit attempts entered into the model. Motivation to quit smoking was only available for people who were smoking at the time of the survey and was therefore not included in the analyses involving quit success.*

This analysis plan was altered after initially running the models as the authors realised that the method of variable selection for these adjusted analyses failed to include important variables. The initial plan to use the univariate analyses of each variable and include those which showed significance in the multivariate analysis ignored the fact that individual variables that are weakly associated with the outcome can contribute significantly when they are combined. Therefore, the authors decided to include all variables in the model as this reduces selection bias and standard errors ^18^, except for those variables that were only included in the survey from 2013 and therefore had substantial missing data (annual household income, ethnicity, highest educational qualification, employment status, marital status, children in household, housing tenure, sexual orientation, disability, mental health diagnoses) to maximise sample size.
