# Supplementary material for: Potential Explanations for Conflicting Findings on Abrupt Versus Gradual Smoking Cessation: A Population Study in England
Source: Nicotine Tob Res. 2021 Nov 18;24(4):574–80. doi: 10.1093/ntr/ntab239 (PMC8887587; doi:10.1093/ntr/ntab239)
Supplement: ntab239_suppl_Supplementary_Tables [file ntab239_suppl_supplementary_tables.docx]

# Supplementary Tables

Supplementary Table 1: Participant characteristics for the whole sample and by type of most recent quit attempt

| *Sociodemographic characteristics* | | | | Whole sample | Abrupt quit attempt | Gradual quit attempt | Association with quit attempt type (abrupt vs. gradual [ref]) | | |
| --- | --- | --- | --- | --- | --- | --- | --- | --- | --- |
|  |  |  |  |  |  |  | OR (95% CI) | p-value | BF_(0,2)_ |
| Age, % (n) | | | |  |  |  |  |  |  |
|  | | 16-24 [ref] | | 20.0 (4,319) | 19.8 (2,341) | 20.4 (1,978) |  |  |  |
|  | | 25-34 | | 23.4 (5,031) | 24.0 (2,840) | 22.6 (2,191) | 1.09 (1.02, 1.16) | .012 | 3.60 |
|  | | 35-44 | | 19.9 (4,282) | 21.1 (2,497) | 18.4 (1,785) | 1.19 (1.11, 1.27) | <.001 | >10,000 |
|  | | 45-54 | | 16.0 (3,441) | 16.1 (1,906) | 15.8 (1,535) | 1.02 (0.95, 1.10) | .526 | 0.09 |
|  | | 55-64 | | 11.7 (2,522) | 10.7 (1,261) | 13.0 (1,261) | 0.80 (0.74, 0.87) | <.001 | >10,000 |
|  | | 65+ | | 9.0 (1,947) | 8.3 (981) | 9.9 (966) | 0.82 (0.75, 0.90) | <.001 | 813 |
| Sex, % (n) | | | |  |  |  |  |  |  |
|  | Male [ref] | | | 48.3 (10,398) | 48.0 (5,678) | 48.6 (4,720) |  |  |  |
|  | Female | | | 51.7 (11,144) | 52.0 (6,148) | 51.4 (4,996) | 1.02 (0.97, 1.08) | .407 | 0.08 |
| Social grade, % (n) | | | |  |  |  |  |  |  |
|  | | AB [ref] | | 12.1 (2,614) | 13.2 (1,561) | 10.8 (1,053) |  |  |  |
|  | | C1 | | 25.3 (5,454) | 25.9 (3,062) | 24.6 (2,392) | 1.07 (1.01, 1.14) | .033 | 0.81 |
|  | | C2 | | 22.5 (4,842) | 22.9 (2,704) | 22.0 (2,138) | 1.05 (0.99, 1.12) | .132 | 0.26 |
|  | | D | | 18.5 (3,994) | 18.1 (2,143) | 19.1 (1,851) | 0.94 (0.88, 1.01) | .081 | 0.42 |
|  | | E | | 21.5 (4,638) | 19.9 (2,356) | 23.5 (2,282) | 0.81 (0.76, 0.86) | <.001 | >10,000 |
| Annual household income, % (n) | | | |  |  |  |  |  |  |
|  | | <£11,499 [ref] | | 28.5 (1,669) | 26.3 (835) | 31.2 (834) |  |  |  |
|  | | £11,500 - £17,499 | | 17.9 (1,045) | 17.5 (556) | 18.3 (489) | 0.95 (0.83, 1.08) | .426 | 0.20 |
|  | | £17,500 - £39,999 | | 31.1 (1,820) | 31.5 (1.002) | 30.6 (818) | 1.04 (0.93, 1.17) | .447 | 0.16 |
|  | | >£40,000 | | 22.5 (1,313) | 24.7 (783) | 19.8 (530) | 1.32 (1.17, 1.50) | <.001 | 1,492 |
| Ethnicity, % (n) | | | |  |  |  |  |  |  |
|  | | | Minority ethnic group [ref] | 12.3 (1,068) | 10.5 (476) | 14.2 (592) |  |  |  |
|  | | | White | 87.7 (7,637) | 89.5 (4,046) | 85.8 (3,591) | 1.40 (1.23, 1.59) | <.001 | >10,000 |
| Educational qualification, % (n) | | | |  |  |  |  |  |  |
|  | | | Post-16 [ref] | 60.4 (5,275) | 62.2 (2,818) | 58.4 (2,457) |  |  |  |
|  | | | Pre-16 | 39.6 (3,463) | 37.8 (1,715) | 41.6 (1,748) | 0.86 (0.79, 0.93) | <.001 | 141 |
| Employment status, % (n) | | | |  |  |  |  |  |  |
|  | | | Not [ref] | 63.9 (5,583) | 62.5 (2,835) | 65.3 (2,748) |  |  |  |
|  | | | Full time job | 36.1 (3,155) | 37.5 (1,698) | 34.6 (1,457) | 1.13 (1.04, 1.23) | .006 | 6.63 |
| Marital status, % (n) | | | |  |  |  |  |  |  |
|  | | | Not [ref] | 53.2 (4,643) | 52.0 (2,352) | 54.6 (2,291) |  |  |  |
|  | | | Married | 46.8 (4,079) | 48.0 (2,174) | 45.4 (1,905) | 1.11 (1.02, 1.21) | .014 | 2.07 |
| Children in household, % (n) | | | |  |  |  |  |  |  |
|  | | | Not [ref] | 63.3 (5,533) | 62.7 (2,841) | 64.0 (2,692) |  |  |  |
|  | | | Present | 36.7 (3,205) | 37.3 (1,692) | 36.0 (1,513) | 1.06 (0.97, 1.16) | .192 | 0.26 |
| Housing tenure, % (n) | | | |  |  |  |  |  |  |
|  | | | Other [ref] | 61.4 (5,257) | 60.2 (2,679) | 62.7 (2,578) |  |  |  |
|  | | | owner occupied | 38.6 (3,303) | 39.8 (1,769) | 37.3 (1,534) | 1.11 (1.02, 1.21) | .019 | 2.07 |
| Sexual orientation, % (n) | | | |  |  |  |  |  |  |
|  | | | Other [ref] | 9.8 (785) | 9.4 (391) | 10.2 (394) |  |  |  |
|  | | | Heterosexual | 90.2 (7,239) | 90.6 (3,772) | 89.8 (3,467) | 1.10 (0.95, 1.27) | .221 | 0.44 |
| Disability, % (n) | | | |  |  |  |  |  |  |
|  | | | No [ref] | 82.4 (6,635) | 83.2 (3,488) | 81.5 (3,147) |  |  |  |
|  | | | Yes | 17.6 (1,420) | 16.8 (706) | 18.5 (714) | 0.89 (0.80, 1.00) | .051 | 1.12 |
| Mental health diagnosis, % (n) | | | |  |  |  |  |  |  |
|  | | | No [ref] | 96.2 (20,729) | 96.2 (11,373) | 96.3 (9,356) |  |  |  |
|  | | | Yes | 3.8 (813) | 3.8 (453) | 3.7 (360) | 1.04 (0.90, 1.19) | .631 | 0.16 |
| *Smoking characteristics* | | | |  |  |  |  |  |  |
| Time to first cigarette, % (n) | | | |  |  |  |  |  |  |
|  | | More than 60 minutes [ref] | | 32.8 (7,021) | 33.9 (3,986) | 31.4 (3,035) |  |  |  |
|  | | 30-60 minutes | | 16.2 (3,476) | 15.4 (1,809) | 17.2 (1,667) | 0.87 (0.81, 0.94) | <.001 | 56.16 |
|  | | 6-30 minutes | | 31.8 (6,807) | 30.8 (3,618) | 33.0 (3,189) | 0.90 (0.85, 0.96) | <.001 | 15.70 |
|  | | Within 5 minutes | | 19.2 (4,112) | 19.9 (2,334) | 18.4 (1,778) | 1.10 (1.03, 1.18) | .006 | 3.52 |
| Strength of urges, % (n) | | | |  |  |  |  |  |  |
|  | | None [ref] | | 15.6 (3,218) | 19.2 (2,173) | 11.2 (1,045) |  |  |  |
|  | | Slight | | 13.6 (2,806) | 13.7 (1,553) | 13.5 (1,253) | 1.02 (0.94, 1.11) | .582 | 0.09 |
|  | | Moderate | | 42.7 (8,800) | 40.0 (4,526) | 46.0 (4,274) | 0.79 (0.74, 0.83) | <.001 | >10,000 |
|  | | Strong | | 19.7 (4,060) | 18.6 (2,105) | 21.0 (1,955) | 0.86 (0.80, 0.92) | <.001 | 1,442 |
|  | | Very strong | | 6.1 (1,262) | 6.0 (674) | 6.3 (588) | 0.94 (0.84, 1.05) | .283 | 0.25 |
|  | | Extremely strong | | 2.2 (461) | 2.4 (275) | 2.0 (186) | 1.22 (1.01, 1.48) | .037 | 2.02 |
| Motivation to quit smoking^a^, mean (SD) | | | | 4.7 (1.73) | 4.6 (1.78) | 4.8 (1.68) | 0.93 (0.92, 0.95) | <.001 | >10,000 |
| Number of quit attempts in past 12 months, % (n) | | | |  |  |  |  |  |  |
|  | | 1 [ref] | | 65.2 (14,054) | 69.4 (8,212) | 60.1 (5,842) |  |  |  |
|  | | 2 | | 21.2 (4,559) | 19.0 (2,244) | 23.8 (2,315) | 0.75 (0.70, 0.80) | <.001 | >10,000 |
|  | | 3 or more | | 13.6 (2,929) | 11.6 (1,370) | 16.0 (1,559) | 0.69 (0.63, 0.74) | <.001 | >10,000 |
| *Quit attempt characteristics* | | | |  |  |  |  |  |  |
| Quit success, % ex-smoker (n) | | | | 15.0 (3,225) | 18.8 (2,224) | 10.3 (1,001) | 2.02 (1.86, 2.19) | <.001 | >10,000 |
| Abrupt quit attempt, % (n) | | | | 54.9 (11,826) | - | - |  | - |  |
| ‘Always abrupt’ quit attempt, % (n) | | | | 51.0 (10,977) | - | - |  | - |  |
| Use of evidence-based aid, % (n) | | | |  |  |  |  |  |  |
|  | | | No [ref] | 46.5 (10,008) | 49.7 (5,882) | 42.5 (4,126) |  |  |  |
|  | | | Yes | 53.5 (11,534) | 50.3 (5,944) | 57.5 (5,590) | 0.75 (0.71, 0.79) | <.001 | >10,000 |
| Type of evidence-based aid used^b^, % (n) | | | |  |  |  |  |  |  |
|  | | Varenicline | | 10.3 (1,184) | 8.4 (497) | 12.3 (687) | 0.65 (0.57, 0.74) | <.001 | >10,000 |
|  | | Bupropion | | 2.9 (334) | 2.5 (148) | 3.3 (186) | 0.74 (0.60, 0.92) | .008 | 11.35 |
|  | | NRT (any type) | | 63.4 (7,312) | 66.0 (3,925) | 60.6 (3,387) | 1.26 (1.17, 1.36) | <.001 | >10,000 |
|  | | E-cigarettes | | 34.5 (2,960) | 34.9 (1,479) | 34.1 (1,481) | 1.03 (0.95, 1.13) | .451 | 0.12 |
|  | | Face-to-face behavioural support | | 8.3 (960) | 8.1 (483) | 8.5 (477) | 0.95 (0.83, 1.08) | .429 | 0.20 |
| Type of NRT used^c^, % over-the-counter (n) | | | | 78.3 (5,547) | 77.6 (2,971) | 79.2 (2,576) | 0.91 (0.81, 1.02) | .114 | 0.58 |
| Time since start of most recent quit attempt, % (n) | | | |  |  |  |  |  |  |
|  | | Last week [ref] | | 5.4 (1,166) | 4.4 (519) | 6.7 (647) |  |  |  |
|  | | Between a week and a month | | 10.9 (2,342) | 9.6 (1,134) | 12.5 (1,208) | 0.75 (0.69, 0.81) | <.001 | >10,000 |
|  | | 1-2 months | | 12.0 (2,571) | 10.9 (1,284) | 13.3 (1,287) | 0.80 (0.73, 0.87) | <.001 | >10,000 |
|  | | 2-3 months | | 12.7 (2,726) | 11.8 (1,388) | 13.8 (1,338) | 0.83 (0.77, 0.90) | <.001 | 3,009 |
|  | | 3-6 months | | 22.2 (4,768) | 23.3 (2,750) | 20.8 (2,018) | 1.16 (1.08, 1.23) | <.001 | >10,000 |
|  | | 6-12 months | | 36.9 (7,924) | 40.1 (4,728) | 33.0 (3,196) | 1.36 (1.28, 1.44) | <.001 | >10,000 |

^a^ Only available for current smokers (1='Not want to stop' and 7='REALLY want to and intend to in the next month')
^b^ if evidence-based aid was used
^c^ if NRT was used

Supplementary Table 2: Participant characteristics for the whole sample and by type of quit attempt, ‘always abrupt’ versus ‘any gradual’ (including measure for daily cigarette consumption)

| *Sociodemographic characteristics* | | Whole sample | ‘Always abrupt’ quit attempts | ‘Any gradual’ quit attempt | Association with quit attempt type (‘always abrupt’ vs. ‘any gradual’ [ref]) | |
| --- | --- | --- | --- | --- | --- | --- |
|  |  |  |  |  | OR (95% CI) | p-value |
| Age, % (n) | |  |  |  |  |  |
|  | 16-24 [ref] | 20.0 (4,319) | 19.4 (2,129) | 20.7 (2,190) |  |  |
|  | 25-34 | 23.4 (5,031) | 24.2 (2,658) | 22.5 (2,373) | 1.10 (1.04, 1.17) | .002 |
|  | 35-44 | 19.9 (4,282) | 21.3 (2,336) | 18.4 (1,946) | 1.20 (1.12, 1.28) | <.001 |
|  | 45-54 | 16.0 (3,441) | 16.1 (1,770) | 15.8 (1,671) | 1.02 (0.95, 1.10) | .537 |
|  | 55-64 | 11.7 (2,522) | 10.6 (1,169) | 12.8 (1,353) | 0.81 (0.75, 0.88) | <.001 |
|  | 65+ | 9.0 (1,947) | 8.3 (915) | 9.8 (1,032) | 0.84 (0.77, 0.92) | <.001 |
| Sex, % female (n) | | 51.7 (11,144) | 51.9 (5,694) | 51.6 (5,450) | 1.01 (0.96, 1.07) | .674 |
| Social grade, % (n) | |  |  |  |  |  |
|  | AB [ref] | 12.1 (2,614) | 13.4 (1,470) | 10.8 (1,144) |  |  |
|  | C1 | 25.3 (5,454) | 26.2 (2,874) | 24.4 (2,580) | 1.10 (1.03, 1.17) | .003 |
|  | C2 | 22.5 (4,842) | 22.9 (2,518) | 22.0 (2,324) | 1.06 (0.99, 1.13) | .098 |
|  | D | 18.5 (3,994) | 17.9 (1,965) | 19.2 (2,029) | 0.92 (0.86, 0.98) | .014 |
|  | E | 21.5 (4,638) | 19.6 (2,150) | 23.5 (2,488) | 0.79 (0.74, 0.84) | <.001 |
| Annual household income, % (n) | |  |  |  |  |  |
|  | <£11,499 [ref] | 28.5 (1,669) | 26.2 (779) | 31.0 (890) |  |  |
|  | £11,500 - £17,499 | 17.9 (1,045) | 17.6 (524) | 18.1 (521) | 0.97 (0.85, 1.10) | .616 |
|  | £17,500 - £39,999 | 31.1 (1,820) | 31.6 (939) | 30.7 (881) | 1.04 (0.93, 1.17) | .443 |
|  | >£40,000 | 22.5 (1,313) | 24.6 (731) | 20.3 (582) | 1.28 (1.14, 1.45) | <.001 |
| Ethnicity, % white (n) | | 87.7 (7,637) | 89.8 (3,800) | 85.7 (3,837) | 1.47 (1.29, 1.67) | <.001 |
| Educational qualification, % pre-16 (n) | | 39.6 (3,463) | 37.5 (1,589) | 41.7 (1,874) | 0.84 (0.77, 0.91) | <.001 |
| Employment status, % full time job (n) | | 36.1 (3,155) | 37.8 (1,604) | 34.5 (1,551) | 1.16 (1.06, 1.26) | .001 |
| Marital status, % married (n) | | 46.8 (4,079) | 48.2 (2,041) | 45.4 (2,038) | 1.12 (1.03, 1.22) | .001 |
| Children in household, % present (n) | | 36.7 (3,205) | 37.3 (1,580) | 36.1 (1,625) | 1.05 (0.96, 1.14) | .278 |
| Housing tenure, % owner occupied (n) | | 38.6 (3,303) | 40.2 (1,674) | 37.0 (1,629) | 1.14 (1.05, 1.25) | .002 |
| Sexual orientation, % heterosexual (n) | | 90.2 (7,239) | 90.7 (3,527) | 89.8 (3,712) | 1.10 (0.95, 1.28) | .187 |
| Disability, % yes (n) | | 17.6 (1,420) | 16.8 (659) | 18.4 (761) | 0.89 (0.80, 1.00) | .055 |
| Mental health diagnosis, % yes (n) | | 3.8 (813) | 3.7 (411) | 3.8 (402) | 0.98 (0.85, 1.13) | .815 |
| *Smoking characteristics* | |  |  |  |  |  |
| Daily cigarette consumption, mean (SD) | | 12.3 (8.60) | 12.9 (8.97) | 11.7 (8.16) | 1.02 (1.01, 1.02) | <.001 |
| Time to first cigarette, % (n) | |  |  |  |  |  |
|  | More than 60 minutes [ref] | 32.8 (7,021) | 34.5 (3,759) | 31.0 (3,262) |  |  |
|  | 30-60 minutes | 16.2 (3,476) | 15.0 (1,634) | 17.5 (1,842) | 0.83 (0.77, 0.89) | <.001 |
|  | 6-30 minutes | 31.8 (6,807) | 30.8 (3,357) | 32.8 (3,450) | 0.91 (0.86, 0.96) | .002 |
|  | Within 5 minutes | 19.2 (4,112) | 19.7 (2,152) | 18.6 (1,960) | 1.07 (1.00, 1.15) | .042 |
| Strength of urges, % (n) | |  |  |  |  |  |
|  | None [ref] | 15.6 (3,218) | 19.6 (2,080) | 11.4 (1,138) |  |  |
|  | Slight | 13.6 (2,806) | 13.9 (1,472) | 13.3 (1,334) | 1.05 (0.97, 1.14) | .234 |
|  | Moderate | 42.7 (8,800) | 39.8 (4,219) | 45.8 (4,581) | 0.78 (0.74, 0.83) | <.001 |
|  | Strong | 19.7 (4,060) | 18.4 (1,950) | 21.1 (2,110) | 0.84 (0.79, 0.90) | <.001 |
|  | Very strong | 6.1 (1,262) | 5.9 (623) | 6.4 (639) | 0.92 (0.82, 1.03) | .133 |
|  | Extremely strong | 2.2 (461) | 2.4 (251) | 2.1 (210) | 1.13 (0.94, 1.36) | .188 |
| Motivation to quit smoking^a^, mean (SD) | | 4.7 (1.73) | 4.6 (1.78) | 4.8 (1.68) | 0.92 (0.91, 0.94) | <.001 |
| Number of quit attempts in past 12 months, % (n) | |  |  |  |  |  |
|  | 1 [ref] | 65.2 (14,054) | 74.8 (8,212) | 55.3 (5,842) |  |  |
|  | 2 | 21.2 (4,559) | 16.3 (1,794) | 26.2 (2,765) | 0.55 (0.52, 0.59) | <.001 |
|  | 3 or more | 13.6 (2,929) | 18.8 (971) | 18.5 (1,958) | 0.43 (0.39, 0.46) | <.001 |
| *Quit attempt characteristics* | |  |  |  |  |  |
| Quit success, % ex-smoker (n) | | 15.0 (3,225) | 19.3 (2,123) | 10.4 (1,102) | 2.06 (1.90, 2.23) | <.001 |
| Abrupt quit attempt, % (n) | | 54.9 (11,826) | - | - |  | - |
| Always abrupt quit attempt, % (n) | | 51.0 (10,977) | - | - |  | - |
| Use of evidence-based aid, % yes (n) | | 53.5 (11,534) | 49.6 (5,446) | 57.6 (6,088) | 0.72 (0.69, 0.76) | <.001 |
| Type of evidence-based aid used^b^, % (n) | |  |  |  |  |  |
|  | Varenicline | 10.3 (1,184) | 8.3 (454) | 12.0 (730) | 0.67 (0.59, 0.75) | <.001 |
|  | Bupropion | 2.9 (334) | 2.4 (129) | 3.4 (205) | 0.70 (0.56, 0.87) | .001 |
|  | NRT (any type) | 63.4 (7,312) | 66.1 (3,599) | 61.0 (3,713) | 1.25 (1.16, 1.35) | <.001 |
|  | E-cigarettes | 34.5 (2,960) | 35.4 (1,365) | 33.7 (1,595) | 1.08 (0.99, 1.18) | .096 |
|  | Face-to-face behavioural support | 8.3 (960) | 8.1 (441) | 8.5 (519) | 0.95 (0.83, 1.08) | .407 |
| Type of NRT used^c^, % over-the-counter (n) | | 78.3 (5,547) | 77.5 (2,717) | 79.1 (2,830) | 0.91 (0.81, 1.02) | .103 |
| Time since start of most recent quit attempt, % (n) | |  |  |  |  |  |
|  | Last week [ref] | 5.4 (1,166) | 4.2 (456) | 6.7 (710) |  |  |
|  | Between a week and a month | 10.9 (2,342) | 9.3 (1,015) | 12.6 (1,327) | 0.71 (0.65, 0.77) | <.001 |
|  | 1-2 months | 12.0 (2,571) | 10.6 (1,162) | 13.4 (1,409) | 0.77 (0.71, 0.83) | <.001 |
|  | 2-3 months | 12.7 (2,726) | 11.3 (1,238) | 14.1 (1,488) | 0.77 (0.71, 0.84) | <.001 |
|  | 3-6 months | 22.2 (4,768) | 23.2 (2,537) | 21.2 (2,231) | 1.12 (1.05, 1.20) | <.001 |
|  | 6-12 months | 36.9 (7,924) | 41.5 (4,550) | 32.0 (3,374) | 1.51 (1.43, 1.59) | <.001 |

^a^ Only available for current smokers (1='Not want to stop' and 7='REALLY want to and intend to in the next month')
^b^ if evidence-based aid was used
^c^ if NRT was used

Supplementary Table 3: Associations with quit attempt type and quit success (n=20,446)

|  | | | Association with quit attempt type (abrupt vs. gradual [ref]) (adjusted) | | | Association with quit success (adjusted) | | |
| --- | --- | --- | --- | --- | --- | --- | --- | --- |
|  | | | OR (95% CI) | p-value | BF_(0,2)_ | OR (95% CI) | p-value | BF_(0,3)_ |
| Quit attempt type (gradual [ref] versus abrupt) | | | - | - | - | 1.75 (1.59, 1.93) | <0.001 | >10,000 |
| Age | | |  |  |  |  |  |  |
|  | 16-24 [ref] | |  |  |  |  |  |  |
|  | 25-34 | | 1.06 (0.98, 1.16) | 0.166 | 0.26 | 1.03 (0.90, 1.19) | 0.665 | 0.10 |
|  | 35-44 | | 1.18 (1.08, 1.29) | <0.001 | 96.01 | 1.08 (0.93, 1.26) | 0.301 | 0.19 |
|  | 45-55 | | 1.08 (0.98, 1.19) | 0.116 | 0.44 | 1.17 (1.00, 1.37) | 0.052 | 0.94 |
|  | 55-64 | | 0.89 (0.80, 0.99) | 0.027 | 1.51 | 1.41 (1.18, 1.67) | <0.001 | 409 |
|  | 65+ | | 0.89 (0.79, 0.99) | 0.038 | 1.51 | 1.63 (1.36, 1.95) | <0.001 | >10,000 |
| Sex | | |  |  |  |  |  |  |
|  | | Male [ref] |  |  |  |  |  |  |
|  | | Female | 1.03 (0.97, 1.09) | 0.298 | 0.12 | 0.99 (0.90, 1.09) | 0.872 | 0.05 |
| Social grade | | |  |  |  |  |  |  |
|  | AB [ref] | |  |  |  |  |  |  |
|  | C1 | | 0.86 (0.78, 0.95) | 0.003 | 11.75 | 0.94 (0.81, 1.08) | 0.375 | 0.15 |
|  | C2 | | 0.87 (0.78, 0.96) | 0.006 | 6.61 | 0.75 (0.64, 0.87) | <0.001 | 180 |
|  | D | | 0.78 (0.70, 0.87) | <0.001 | 3,137 | 0.70 (0.60, 0.83) | <0.001 | 678 |
|  | E | | 0.72 (0.65, 0.80) | <0.001 | >10,000 | 0.63 (0.53, 0.74) | <0.001 | >10,000 |
| Time to first cigarette | | |  |  |  |  |  |  |
|  | More than 60 minutes [ref] | |  |  |  |  |  |  |
|  | 30-60 minutes | | 0.92 (0.84, 1.00) | 0.047 | 0.81 | 1.23 (1.07, 1.43) | 0.005 | 5.13 |
|  | 6-30 minutes | | 0.99 (0.92, 1.06) | 0.755 | 0.06 | 1.37 (1.22, 1.55) | <0.001 | >10,000 |
|  | Within 5 minutes | | 1.17 (1.07, 1.28) | <0.001 | 45.39 | 1.54 (1.33, 1.78) | <0.001 | >10,000 |
| Strength of urges, % (n) | | |  |  |  |  |  |  |
|  | None [ref] | |  |  |  |  |  |  |
|  | Slight | | 0.63 (0.57, 0.71) | <0.001 | >10,000 | 0.13 (0.11, 0.15) | <0.001 | >10,000 |
|  | Moderate | | 0.56 (0.52, 0.61) | <0.001 | >10,000 | 0.05 (0.04, 0.05) | <0.001 | >10,000 |
|  | Strong | | 0.59 (0.53, 0.65) | <0.001 | >10,000 | 0.04 (0.03, 0.04) | <0.001 | >10,000 |
|  | Very strong | | 0.63 (0.55, 0.72) | <0.001 | >10,000 | 0.05 (0.04, 0.06) | <0.001 | >10,000 |
|  | Extremely strong | | 0.81 (0.66, 1.00) | 0.049 | 1.95 | 0.05 (0.03, 0.07) | <0.001 | >10,000 |
| Number of quit attempts in past 12 months | | |  |  |  |  |  |  |
|  | 1 [ref] | |  |  |  |  |  |  |
|  | 2 | | 0.76 (0.71, 0.82) | <0.001 | >10,000 | 0.48 (0.42, 0.55) | <0.001 | >10,000 |
|  | 3 or more | | 0.73 (0.67, 0.80) | <0.001 | >10,000 | 0.37 (0.32, 0.44) | <0.001 | >10,000 |
| Use of evidence-based aid | | |  |  |  |  |  |  |
|  | | no [ref] |  |  |  |  |  |  |
|  | | yes | 0.76 (0.72, 0.81) | <0.001 | >10,000 | 1.30 (1.18, 1.43) | <0.001 | >10,000 |
| Time since start of most recent quit attempt | | |  |  |  |  |  |  |
|  | Last week [ref] | |  |  |  |  |  |  |
|  | Between a week and a month | | 1.19 (1.03, 1.38) | 0.018 | 2.94 | 0.50 (0.41, 0.61) | <0.001 | >10,000 |
|  | 1-2 months | | 1.27 (1.10, 1.47) | 0.001 | 34.18 | 0.23 (0.18, 0.28) | <0.001 | >10,000 |
|  | 2-3 months | | 1.32 (1.15, 1.53) | <0.001 | 178.22 | 0.18 (0.14, 0.22) | <0.001 | >10,000 |
|  | 3-6 months | | 1.68 (1.47, 1.92) | <0.001 | >10,000 | 0.18 (0.15, 0.22) | <0.001 | >10,000 |
|  | 6-12 months | | 1.69 (1.48, 1.92) | <0.001 | >10,000 | 0.17 (0.14, 0.20) | <0.001 | >10,000 |

Supplementary Table 4: Associations with quit attempt type (‘always abrupt’ versus ‘any gradual’) and quit success (n=20,446)

|  | | | Association with quit attempt type (‘always abrupt’ vs. ‘any gradual’ [ref]), (adjusted) | | | Association with quit status (adjusted) | | |
| --- | --- | --- | --- | --- | --- | --- | --- | --- |
|  | | | OR (95% CI) | p-value | BF_(0,2)_ | OR (95% CI) | p-value | BF_(0,3)_ |
| Quit attempt type (‘any gradual’ [ref] versus ‘always abrupt’) | | | - | - | - | 1.66 (1.51, 1.83) | <0.001 | >10,000 |
| Age | | |  |  |  |  |  |  |
|  | 16-24 [ref] | |  |  |  |  |  |  |
|  | 25-34 | | 1.12 (1.02, 1.22) | 0.012 | 3.60 | 1.03 (0.89, 1.19) | 0.676 | 0.10 |
|  | 35-44 | | 1.25 (1.14, 1.37) | <0.001 | >10,000 | 1.08 (0.93, 1.25) | 0.309 | 0.20 |
|  | 45-55 | | 1.13 (1.03, 1.24) | 0.012 | 3.72 | 1.17 (1.00, 1.37) | 0.053 | 0.94 |
|  | 55-64 | | 0.93 (0.84, 1.04) | 0.209 | 0.33 | 1.40 (1.18, 1.66) | <0.001 | 270 |
|  | 65+ | | 0.97 (0.86, 1.09) | 0.607 | 0.13 | 1.62 (1.35, 1.94) | <0.001 | >10,000 |
| Sex | | |  |  |  |  |  |  |
|  | | male [ref] |  |  |  |  |  |  |
|  | | female | 1.01 (0.96, 1.07) | 0.692 | 0.05 | 0.99 (0.91, 1.09) | 0.914 | 0.05 |
| Social grade | | |  |  |  |  |  |  |
|  | AB [ref] | |  |  |  |  |  |  |
|  | C1 | | 0.86 (0.78, 0.95) | 0.004 | 11.75 | 0.93 (0.81, 1.08) | 0.367 | 0.18 |
|  | C2 | | 0.87 (0.78, 0.96) | 0.005 | 6.61 | 0.75 (0.64, 0.87) | <0.001 | 180 |
|  | D | | 0.77 (0.69, 0.86) | <0.001 | 6,977 | 0.71 (0.60, 0.83) | <0.001 | 1,415 |
|  | E | | 0.72 (0.65, 0.80) | <0.001 | >10,000 | 0.63 (0.53, 0.74) | <0.001 | >10,000 |
| Time to first cigarette | | |  |  |  |  |  |  |
|  | More than 60 minutes [ref] | |  |  |  |  |  |  |
|  | 30-60 minutes | | 0.87 (0.80, 0.95) | 0.002 | 15.62 | 1.24 (1.07, 1.43) | 0.004 | 10.24 |
|  | 6-30 minutes | | 0.97 (0.90, 1.04) | 0.408 | 0.12 | 1.38 (1.22, 1.55) | <0.001 | >10,000 |
|  | Within 5 minutes | | 1.15 (1.05, 1.26) | 0.002 | 11.80 | 1.55 (1.34, 1.78) | <0.001 | >10,000 |
| Strength of urges, % (n) | | |  |  |  |  |  |  |
|  | None [ref] | |  |  |  |  |  |  |
|  | Slight | | 0.65 (0.59, 0.73) | <0.001 | >10,000 | 0.13 (0.11, 0.15) | <0.001 | >10,000 |
|  | Moderate | | 0.58 (0.53, 0.63) | <0.001 | >10,000 | 0.05 (0.04, 0.05) | <0.001 | >10,000 |
|  | Strong | | 0.60 (0.55, 0.67) | <0.001 | >10,000 | 0.04 (0.03, 0.04) | <0.001 | >10,000 |
|  | Very strong | | 0.66 (0.57, 0.75) | <0.001 | >10,000 | 0.05 (0.04, 0.06) | <0.001 | >10,000 |
|  | Extremely strong | | 0.81 (0.66, 1.00) | 0.045 | 1.95 | 0.05 (0.03, 0.07) | <0.001 | >10,000 |
| Number of quit attempts in past 12 months | | |  |  |  |  |  |  |
|  | 1 [ref] | |  |  |  |  |  |  |
|  | 2 | | 0.53 (0.49, 0.57) | <0.001 | >10,000 | 0.50 (0.44, 0.57) | <0.001 | >10,000 |
|  | 3 or more | | 0.43 (0.39, 0.47) | <0.001 | >10,000 | 0.40 (0.34, 0.47) | <0.001 | >10,000 |
| Use of evidence-based aid | | |  |  |  |  |  |  |
|  | | no [ref] |  |  |  |  |  |  |
|  | | yes | 0.73 (0.69, 0.78) | <0.001 | >10,000 | 1.30 (1.18, 1.43) | <0.001 | >10,000 |
| Time since start of most recent quit attempt | | |  |  |  |  |  |  |
|  | Last week [ref] | |  |  |  |  |  |  |
|  | Between a week and a month | | 1.23 (1.06, 1.43) | 0.008 | 7.91 | 0.50 (0.41, 0.61) | <0.001 | >10,000 |
|  | 1-2 months | | 1.35 (1.17, 1.57) | <0.001 | 399.11 | 0.23 (0.18, 0.28) | <0.001 | >10,000 |
|  | 2-3 months | | 1.33 (1.14, 1.54) | <0.001 | 283.55 | 0.18 (0.14, 0.22) | <0.001 | >10,000 |
|  | 3-6 months | | 1.67 (1.45, 1.92) | <0.001 | >10,000 | 0.19 (0.15, 0.22) | <0.001 | >10,000 |
|  | 6-12 months | | 1.67 (1.46, 1.91) | <0.001 | >10,000 | 0.17 (0.14, 0.20) | <0.001 | >10,000 |

Supplementary Table 5: Participant characteristics for the whole sample and by type of most recent quit attempt, gradual versus abrupt for daily cigarette consumption measure

|  | Whole sample | Abrupt quit attempt | Gradual quit attempt | Association with quit attempt type (abrupt vs. gradual [ref]) | |
| --- | --- | --- | --- | --- | --- |
|  |  |  |  | OR (95% CI) | p-value |
| Daily cigarette consumption, mean (SD) | 12.3 (8.60) | 12.9 (8.98) | 11.6 (8.07) | 1.02 (1.01, 1.02) | <.001 |

Supplementary Table 6: Associations with quit attempt type and quit success (including measure for daily cigarette consumption; n=19,909)

|  | | | Association with quit attempt type (abrupt vs. gradual [ref]) (adjusted) | | | Association with quit success (adjusted) | | |
| --- | --- | --- | --- | --- | --- | --- | --- | --- |
|  | | | OR (95% CI) | p-value | BF_(0,2)_ | OR (95% CI) | p-value | BF_(0,3)_ |
| Quit attempt type (gradual [ref] versus abrupt) | | | - | - | - | 1.63 (1.47, 1.80) | <0.001 | >10,000 |
| Age | | |  |  |  |  |  |  |
|  | 16-24 [ref] | |  |  |  |  |  |  |
|  | 25-34 | | 1.05 (0.96, 1.14) | 0.289 | 0.21 | 0.99 (0.86, 1.15) | 0.931 | 0.08 |
|  | 35-44 | | 1.14 (1.04, 1.25) | 0.006 | 6.47 | 1.03 (0.88, 1.19) | 0.752 | 0.10 |
|  | 45-55 | | 1.01 (0.92, 1.11) | 0.820 | 0.08 | 1.02 (0.86, 1.20) | 0.827 | 0.09 |
|  | 55-64 | | 0.82 (0.74, 0.92) | <0.001 | 49.15 | 1.25 (1.05, 1.50) | 0.013 | 2.91 |
|  | 65+ | | 0.84 (0.74, 0.94) | 0.002 | 16.17 | 1.45 (1.20, 1.75) | <0.001 | 296 |
| Sex | | |  |  |  |  |  |  |
|  | | male [ref] |  |  |  |  |  |  |
|  | | female | 1.08 (1.02, 1.15) | 0.007 | 1.63 | 1.07 (0.97, 1.17) | 0.188 | 0.23 |
| Social grade | | |  |  |  |  |  |  |
|  | AB [ref] | |  |  |  |  |  |  |
|  | C1 | | 0.84 (0.76, 0.93) | 0.001 | 40.67 | 0.93 (0.80, 1.08) | 0.335 | 0.18 |
|  | C2 | | 0.84 (0.76, 0.94) | 0.001 | 16.17 | 0.71 (0.61, 0.84) | <0.001 | 428 |
|  | D | | 0.76 (0.69, 0.85) | <0.001 | >10,000 | 0.67 (0.57, 0.80) | <0.001 | 2,753 |
|  | E | | 0.70 (0.63, 0.77) | <0.001 | >10,000 | 0.59 (0.49, 0.70) | <0.001 | >10,000 |
| Cigarettes per day | | | 1.03 (1.02, 1.03) | <0.001 | >10,000 | 1.05 (1.04, 1.05) | <0.001 | >10,000 |
| Time to first cigarette | | |  |  |  |  |  |  |
|  | More than 60 minutes [ref] | |  |  |  |  |  |  |
|  | 30-60 minutes | | 0.83 (0.76, 0.91) | <0.001 | 344 | 1.06 (0.91, 1.23) | 0.459 | 0.15 |
|  | 6-30 minutes | | 0.83 (0.77, 0.90) | <0.001 | 3,009 | 1.04 (0.91, 1.18) | 0.590 | 0.10 |
|  | Within 5 minutes | | 0.91 (0.83, 1.00) | 0.055 | 0.91 | 0.95 (0.81, 1.12) | 0.558 | 0.14 |
| Strength of urges, % (n) | | |  |  |  |  |  |  |
|  | None [ref] | |  |  |  |  |  |  |
|  | Slight | | 0.66 (0.59, 0.74) | <0.001 | >10,000 | 0.13 (0.11, 0.15) | <0.001 | >10,000 |
|  | Moderate | | 0.57 (0.52, 0.62) | <0.001 | >10,000 | 0.05 (0.04, 0.05) | <0.001 | >10,000 |
|  | Strong | | 0.56 (0.50, 0.62) | <0.001 | >10,000 | 0.03 (0.03, 0.04) | <0.001 | >10,000 |
|  | Very strong | | 0.57 (0.49, 0.66) | <0.001 | >10,000 | 0.04 (0.03, 0.05) | <0.001 | >10,000 |
|  | Extremely strong | | 0.71 (0.58, 0.88) | 0.002 | 36.87 | 0.03 (0.02, 0.05) | <0.001 | >10,000 |
| Use of evidence-based aid | | |  |  |  |  |  |  |
|  | | no [ref] |  |  |  |  |  |  |
|  | | yes | 0.74 (0.70, 0.79) | <0.001 | >10,000 | 1.19 (1.08, 1.32) | <0.001 | 21.13 |
| Time since start of most recent quit attempt | | |  |  |  |  |  |  |
|  | Last week [ref] | |  |  |  |  |  |  |
|  | Between a week and a month | | 1.18 (1.02, 1.37) | 0.030 | 2.22 | 0.48 (0.39, 0.59) | <0.001 | >10,000 |
|  | 1-2 months | | 1.26 (1.09, 1.46) | 0.002 | 23.07 | 0.22 (0.18, 0.28) | <0.001 | >10,000 |
|  | 2-3 months | | 1.32 (1.14, 1.53) | <0.001 | 178 | 0.17 (0.14, 0.21) | <0.001 | >10,000 |
|  | 3-6 months | | 1.64 (1.43, 1.89) | <0.001 | >10,000 | 0.17 (0.14, 0.21) | <0.001 | >10,000 |
|  | 6-12 months | | 1.67 (1.46, 1.91) | <0.001 | >10,000 | 0.15 (0.13, 0.19) | <0.001 | >10,000 |
| Number of quit attempts in past 12 months | | |  |  |  |  |  |  |
|  | 1 [ref] | |  |  |  |  |  |  |
|  | 2 | | 0.78 (0.72, 0.83) | <0.001 | >10,000 | 0.49 (0.43, 0.56) | <0.001 | >10,000 |
|  | 3 or more | | 0.74 (0.68, 0.81) | <0.001 | >10,000 | 0.39 (0.33, 0.46) | <0.001 | >10,000 |
